# Systematic Analysis of Competing Endogenous RNA Networks in Diffuse Large B-Cell Lymphoma and Hodgkin’s Lymphoma

**DOI:** 10.3389/fgene.2020.586688

**Published:** 2020-09-30

**Authors:** Juanjuan Kang, Pengcheng Yao, Qiang Tang, Ying Wang, Yuwei Zhou, Jian Huang

**Affiliations:** ^1^Center for Informational Biology, University of Electronic Science and Technology of China, Chengdu, China; ^2^Affiliated Foshan Maternity & Child Healthcare Hospital, Southern Medical University, Foshan, China; ^3^Innovative Institute of Chinese Medicine and Pharmacy, Chengdu University of Traditional Chinese Medicine, Chengdu, China

**Keywords:** lymphoma, diffuse large B-cell lymphoma, Hodgkin’s lymphoma, competing endogenous RNA, regulatory network

## Abstract

Lymphoma is a systemic malignancy, originating from the lymphatic system, which accounts for 3 to 4% of all tumors. There are two major subtypes of lymphoma, namely, diffuse large B-cell lymphoma (DLBCL) and Hodgkin’s lymphoma (HL). Elucidation of the pathogenesis of these two lymphoma types is crucial for the identification of potential therapeutic targets. Compared with the corresponding knowledge of other diseases, the understanding of the regulatory networks involved in DLBCL and HL is relatively deficient. To address this, we comprehensively analyzed the mRNAs, lncRNAs, and miRNAs that were differentially expressed between normal and tumor samples of DLBCL and HL. In addition, functional enrichment analysis of the differentially expressed mRNAs was performed. We constructed two specific ceRNA networks of DLBCL and HL. The pathways enriched by dysregulated mRNAs in DLBCL and HL were mainly involved in immune responses, transcription process, and metabolism process. The ceRNA network analysis revealed that 45 ceRNAs were shared between the two ceRNA networks, including five pivotal lncRNAs (*MALAT1*, *CTBP1-AS*, *THUMPD3-AS*, *PSMA3-AS1*, and *NUTM2A-AS1*). In addition, we proposed a DLBCL survival risk model based on a DLBCL-specific network constructed by Lasso regression analysis. The model, which is based on eight mRNAs, exhibited excellent performance in regard to predicting outcomes in DLBCL patients, with a *p* value of 0.0017 and AUC of 0.9783. In summary, although the molecular mechanisms underlying tumorigenesis in DLBCL and HL were quite different, the same pivotal lncRNAs acted as key regulators. Our findings identify novel potential prognostic and therapeutic targets for DLBCL and HL.

## Introduction

Lymphoma is a systemic malignancy, arising in the lymphatic system, that accounts for about 3–4% of all tumors. It can be traditionally divided into two major pathological forms: Hodgkin’s lymphoma (HL) and non-Hodgkin’s lymphoma (NHL) (Mugnaini and Ghosh, 2016). Worldwide, nearly 510,000 new cases of NHL were estimated in 2018, with approximately 250,000 deaths, representing 2.8% and 2.6% of all tumors. At the same time, nearly 80,000 new cases (0.4% of all tumors) and 26,000 deaths (0.3% of all tumors) were reported for HL (Bray et al., 2018). Diffuse large B-cell lymphoma (DLBCL), which originates in the germinal center, is the most prevalent subtype of NHL and accounts for 24% of new cases of this form (Liu and Barta, 2019). Even though investigations of DLBCL and HL have resulted in encouraging progress in many important areas, particularly in the identification of driver genes of DLBCL (Reddy et al., 2017) and an improved understanding of HL pathogenesis (Mathas et al., 2016), studies on the regulatory networks involved in the tumorigenesis of DLBCL and HL are lacking. Moreover, there are significant variations between DLBCL patients in regard to survival. Therefore, it is necessary to identify new diagnostic and prognostic biomarkers.

Advances in next-generation sequencing technologies have resulted in the discovery of non-coding RNAs, including micro RNAs (miRNAs, with a length of ∼22 nucleotides) and long non-coding RNAs (lncRNAs, longer than 200 nt in length), as important regulatory molecules. Functional studies have shown that miRNAs and lncRNAs participate in regulating a broad range of biological processes, such as cell differentiation and development (Di Lisio et al., 2012; Fatica and Bozzoni, 2014). The changes in their expression are associated with many human pathologies including, but not limited to, lymphoma (Sole et al., 2017; Zhou et al., 2017). For example, the aberrant downregulation of miR-150 activates the PI3K-AKT pathway, inducing telomerase activation and immortalization of malignant lymphoma cells (Watanabe et al., 2011). Yan et al. found that the lncRNA *HOTAIR* acts as an oncogene whose knockdown leads to cell cycle arrest and the induction of apoptosis in DLBCL; further, its higher expression was found to be associated with poorer prognosis (Yan et al., 2016). Although the role of lncRNAs in DLBCL has been relatively well described (Zhou et al., 2017), their biological functions in HL have not been fully elucidated.

The competing endogenous RNA (ceRNA) hypothesis proposed by Salmena et al. (Salmena et al., 2011) provides a new angle on the potential functions of lncRNAs. According to this hypothesis, mRNA and lncRNA can cross talk with each other through the competitive binding of miRNA. On the basis of this hypothesis, ceRNA networks have been constructed to elucidate pathological mechanisms and identify biomarkers in various types of tumors such as squamous cell carcinoma (Zhou R.S. et al., 2019), myeloid leukemia (Sen et al., 2018), gastric cancer (Zhu et al., 2019), and so on (Xu et al., 2015, 2016; Li et al., 2018). Thus, a delineation of malignant lymphoma from the aspect of ceRNA networks is practicable and necessary.

In this study, we identified differentially expressed lncRNAs, miRNAs, and mRNAs involved in the pathogenesis of DLBCL and HL. Gene Ontology (GO) and Kyoto Encyclopedia of Genes and Genomes (KEGG) enrichment analyses were performed to investigate the potential biological functions of dysregulated genes. Then, two ceRNA networks for the two lymphoma types were constructed. The shared ceRNA pairs across the two types of lymphoma were analyzed to derive new lncRNA biomarkers for DLBCL and HL. In addition, we constructed a survival risk model to stratify patients with DLBCL according to survival.

## Materials and Methods

### Microarray Data

Two publicly available microarray expression profiling datasets consisting of gene expression data for DLBCL and HL were downloaded from the Gene Expression Omnibus (GEO) database. The DLBCL GSE56315 dataset (Dybkaer et al., 2015) and HL dataset of GSE25986 (Steidl et al., 2011) were obtained using the HG-U133 Plus 2.0 expression array from Affymetrix. The samples in the GSE56315 dataset were derived from 55 patients diagnosed with DLBCL and 33 B cells from normal human tonsil samples. The GSE25986 dataset contained data for five samples of both commonly used HL cell lines and reactive germinal center (GC) cell lines, respectively.

We searched the GEO database for all available miRNA expression profiling datasets related to DLBCL and HL and identified two relevant datasets: one dataset consisted of 45 DLBCL and 10 normal lymph node samples in frozen biopsies (accession number GSE42906) (Caramuta et al., 2013); the second dataset (accession number, GSE45264) contained data for eight normal lymph node samples and 14 classical HL lymph node samples in formalin-fixed paraffin-embedded tissue. The microarray expression profiling datasets are summarized in Table 1.

**TABLE 1:** Summary of the microarray expression profiling datasets.

Date type	Accession number	Lymphoma type	Tumor samples	Normal samples
Gene expression profiling	GSE56315	DLBCL	55	33
	GSE25986	HL	5	5
miRNA expression profiling	GSE42906	DLBCL	45	10
	GSE45264	HL	14	8

### Data Processing

The “affy” package in R was used to process the raw CEL files of these two gene datasets. Probe names were annotated with gene IDs according to platform annotation files. After excluding probes without gene IDs or with more than one gene ID, the average values for multiple probes mapped to the same gene IDs were calculated. For miRNA datasets, we downloaded the express matrix directly for further analysis.

We identified differentially expressed RNAs by using the “samr” package in R. Genes with | log_2_FC| ≥ 1 and *q* < 0.01 were considered significantly differentially expressed genes; | log_2_FC| ≥ 1 and *q* < 0.05 for GSE45264 and only *q* < 0.05 for GSE42906 were the cutoff values for differentially expressed miRNAs.

### LncRNA Identification Pipeline

In order to evaluate the expression pattern of lncRNAs in microarray data, we identified the probe sets mapped to lncRNAs from gene expression profiling using the following criteria: (1) We selected 44135 probes with Ensemble gene IDs. (2) Among these, probes annotated with “non-protein coding,” “non-coding,” or “antisense RNA” were saved. (3) To improve the accuracy of recognition, probes that were annotated with microRNA, miRNA, rRNA, tRNA, snRNA, or snoRNA were excluded. Finally, 1867 probes mapping to lncRNAs were identified. The genes mapped by these probes were lncRNA genes, and the remaining were mRNA genes.

### Functional Enrichment Analysis

Gene Ontology and KEGG enrichment analyses were employed to determine the biological functions of the differentially expressed mRNAs in DLBCL and HL. We used Metascape (Zhou Y. et al., 2019)^1^ to conduct functional enrichment analysis. Terms with *p* value < 0.01, minimum count of 3, and enrichment factor >1.5 were collected and grouped into clusters based on their membership similarities. The most statistically significant term within a cluster was chosen to represent the cluster. Heatmaps were constructed with the top 20 enriched GO biological processes and KEGG clusters to analyze the functional differences between differentially expressed mRNAs in DLBCL and HL.

### Identification of miRNA Targets

Based on the ceRNA hypothesis, ceRNA pairs should have shared miRNA targets. The mRNA targets of dysregulated miRNAs were identified using ENCORI, a new version of starBase (Li et al., 2014). The targets should be predicted by at least four programs out of seven (PITA, RNA22, miRmap, DIANA-microT, miRanda, PicTar, and TargetScan). StarBase was used to identify lncRNA targets.

### Construction of the ceRNA Network

There are two necessary conditions that potential lncRNA and mRNA pairs must meet for the construction of a ceRNA network.

Firstly, they should regulate the same miRNA, to a statistically significant extent. We used hypergeometric cumulative distribution function tests to identify candidate lncRNA and mRNA pairs. A *p* value was calculated for each pair using the formula,

(1) $$p=1-F\left(k|A,M,N\right)=1-\sum_{i=0}^{k-1}\frac{\left(\begin{array}{c} M\\ i \end{array}\right)\,\left(\begin{array}{c} A-M\\ N-i \end{array}\right)}{\left(\begin{array}{c} A\\ N \end{array}\right)}\;\left(k>3\right)$$

where *k* is the number of target miRNAs shared with RNA_1_ and RNA_2_; *M* and *N* are the number of target miRNAs of RNA_1_ and RNA2, respectively; and *A* is the total number of dysregulated miRNAs. The Benjamini–Hochberg adjusted *p* value < 0.05 was used to filter out the potential lncRNA and mRNA pairs.

Secondly, the lncRNA and mRNA pairs should exhibit a co-expression pattern. For potential lncRNA and mRNA pairs, we calculated the Pearson correlation coefficient (*R*) based on their expression profiling; *R* > 0.7 were applied to select the candidate lncRNA and mRNA pairs.

Finally, Cytoscape 3.7.2 was used to visualize the DLBCL- and HL-specific ceRNA networks.

### Collection of mRNAs, lncRNAs, and miRNAs Associated With DLBCL and HL

A list of published mRNAs, lncRNAs, and miRNAs related to lymphoma was collated from the state-of-the-art databases. A list of mRNAs associated with DLBCL and HL was collected from DisGeNET v2019 (Pinero et al., 2020). A total of 574 DLBCL-related and 645 HL-related mRNAs was identified by searching the database. A list of lncRNAs associated with the two types of lymphoma was derived from the MNDR v2.0 (Huang et al., 2019), LncRNADisease 2.0 (Bao et al., 2019), and Lnc2Cancer 2.0 (Gao et al., 2019) databases. In the three databases, 15 lncRNAs were related to DLBCL, while only one lncRNA, *PVT1*, was related to HL and also included in the list of lncRNAs associated with DLBCL (Supplementary Table S1). In addition, a list of miRNAs associated with the two types of lymphoma was obtained from HMDD v3.0 (Cui et al., 2018). A list of miRNAs containing 53 DLBCL- and 29 HL-related miRNA precursors was constructed.

### Survival Analysis

To assess the prognostic characteristics of RNAs in the DLBCL-specific ceRNA network, we obtained survival and expression data for the DLBCL samples from the TCGA database and used the “survival” package of R to perform Kaplan–Meier curve analysis. Samples were divided into higher-expression and lower-expression groups based on the median expression level of each RNA. Then, Lasso regression analysis for the significant RNAs was performed to build the prognostic risk model. The performance of the model was evaluated by the area under the receiver operating characteristic curve (AUC) based on 5-year survival outcome.

## Results

### Identification of Differentially Expressed lncRNAs, mRNAs, and miRNAs

**FIGURE 1:**
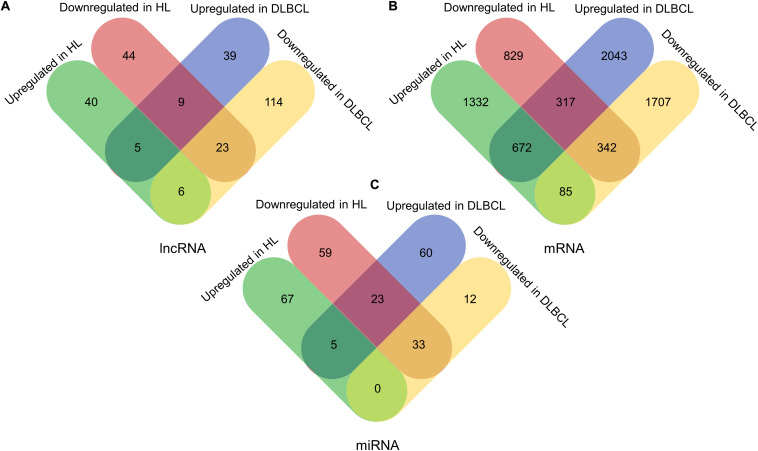
The differentially expressed lncRNAs, mRNAs, and miRNAs in DLBCL and HL. **(A)** The differentially expressed lncRNAs, in DLBCL and HL. **(B)** The differentially expressed mRNAs, in DLBCL and HL. **(C)** The differentially expressed miRNAs, in DLBCL and HL.

In total, 196 lncRNAs (143 downregulated and 53 upregulated lncRNAs) and 127 lncRNAs (76 downregulated and 51 upregulated lncRNAs) were significantly differentially expressed in DLBCL and HL, respectively. In addition, 3032 upregulated and 2134 downregulated mRNAs in DLBCL, and 2089 upregulated and 1488 downregulated mRNAs in HL, were found. Additionally, we identified 133 dysregulated miRNAs (45 downregulated and 88 upregulated) in DLBCL and 187 dysregulated (115 downregulated and 72 upregulated) miRNAs in HL. The dysregulated RNAs are summarized in Table 2. Among them, 43 lncRNAs, 1416 mRNAs, and 61 miRNAs were shared between the two types of lymphoma (Figure 1).

**TABLE 2:** Summary of dysregulated mRNA, lncRNA, and miRNA.

Lymphoma type	Date type	RNA type	Up*	Down*	Total
DLBCL	Gene expression profiling	mRNA	3032	2134	5166
		lncRNA	53	143	196
	miRNA expression profiling	miRNA	88	45	133
HL	Gene expression profiling	mRNA	2089	1488	3577
		lncRNA	51	76	127
	miRNA expression profiling	miRNA	72	115	187

*Up stands for the upregulated RNAs. Down represents the downregulated RNAs.

Compared with the list of published mRNAs, lncRNAs, and miRNAs related to lymphoma, 190 mRNAs and 24 miRNA precursors were dysregulated in DLBCL, and 164 mRNAs and 14 miRNA precursors were dysregulated in HL. Some typical biomarkers of lymphoma were included, such as the five miRNA biomarkers (miR-150, miR-21, miR-155, miR-17, and miR-146a) for distinguishing lymphoma from reactive lymphoid hyperplasia (Kang et al., 2020) and the T-cell lineage protein GATA3 (Atayar et al., 2005).

### GO and KEGG Enrichment Analyses of Differentially Expressed mRNAs

**FIGURE 2:**
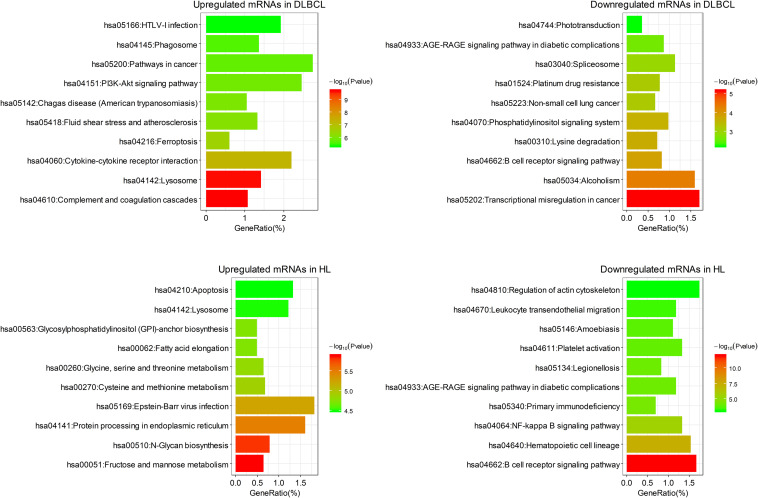
The top 10 clusters of KEGG pathway enriched by differentially expressed mRNAs in DLBCL and HL.

Functional enrichment analysis of the differentially expressed mRNAs was performed. These mRNAs were enriched in pathways previously reported to be associated with B cell lymphoma, such as the PI3K-Akt signaling pathway (Uddin et al., 2006; Yuan et al., 2019) and the NF-κB signaling pathway (Weniger and Kuppers, 2016; Figure 2). In addition, infection with the Epstein–Barr virus has been shown to be highly correlated with pathogenesis of HL, and it affects the survival of subgroups of this form of lymphoma (Myriam et al., 2017). Consistent with this finding, the Epstein–Barr virus infection pathway was enriched in the upregulated mRNAs in HL. Interestingly, downregulated mRNAs in HL were enriched in the B-cell receptor signaling pathway, reflecting the defective B-cell program in classical HL (Kuppers, 2009).

Among the enriched pathways, the upregulated mRNAs in DLBCL were mainly related to parasitic or viral infection, such as cytokine–cytokine receptor interaction and parasitic infection, and the downregulated mRNAs in DLBCL were enriched in transcription pathways. The upregulated mRNAs in HL were mainly involved in pathways associated with metabolism and biosynthesis, and the downregulated mRNAs in HL were enriched in signal transduction (Figure 2).

**FIGURE 3:**
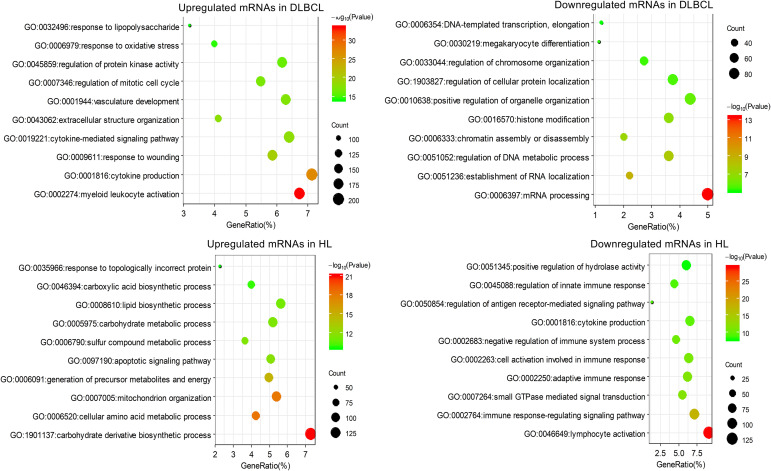
The top 10 clusters of the enriched GO biological process terms for differentially expressed mRNAs in DLBCL and HL.

The enrichment results for GO biological process were similar to that for the KEGG pathway analysis. The upregulated mRNAs in DLBCL were mainly involved in myeloid leukocyte activation, cytokine production, response to wounding, etc. The downregulated mRNAs in DLBCL were mainly involved in transcriptional processes such as mRNA processing and RNA splicing and localization. The mRNAs upregulated in HL mainly participated in a large number of metabolic and biosynthetic processes, including carbohydrate derivative biosynthetic process, cellular amino acid metabolic process, and cofactor metabolic process, whereas the mRNAs that were downregulated in HL were mainly involved in biological processes related to the lymphocyte immune response (Figure 3).

The results of functional enrichment analyses show that there was a significant difference between DLBCL and HL. However, hierarchical cluster analysis of the top 20 enriched pathways and biological processes revealed that the GO terms and KEGG pathways enriched in the mRNAs with the same direction of dysregulation were much more similar than those enriched by the miRNAs of opposite direction of dysregulation in DLBCL and HL (Supplementary Figure S1).

At the same time, we also analyzed the enrichment pathways and biological processes of commonly dysregulated mRNAs in DLBCL and HL (Supplementary Figure S2). The mRNAs that were downregulated in both lymphoma subtypes mainly participated in the biological processes of B cell activation and immunity. The upregulated mRNAs were significantly enriched in metabolism and biosynthesis process, such as cofactor metabolic process, cellular acid metabolic process, and small-molecule catabolic process. The result indicated that the biological processes of metabolism and biosynthesis are simultaneously activated in DLBCL and HL, providing nutrition and energy for tumor cells to promote the continuous proliferation of tumor cells.

### Topological Properties of the ceRNA Networks

**FIGURE 4:**
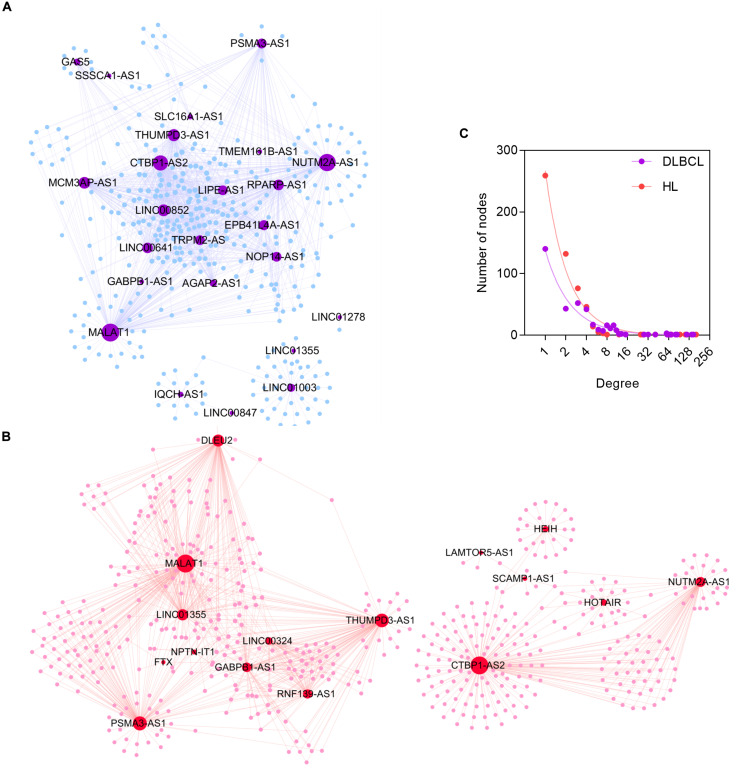
The topological properties of DLBCL- and HL-specific ceRNA networks. **(A)** The DLBCL ceRNA network. **(B)** The HL ceRNA network. **(C)** The node degree distributions of DLBCL- and HL-specific ceRNA networks.

After the filter process based on shared targeted miRNA and co-expression pattern as mentioned in Methods, we constructed DLBCL- and HL-specific ceRNA networks separately. The DLBCL-specific ceRNA network comprised 24 lncRNA nodes, 358 mRNA nodes, and 1237 edges (Figure 4A). The average node degree of mRNA was 3.46 (95% CI: 3.14–3.77): that is, one mRNA had an indirect regulatory relationship with approximately three lncRNAs. The HL-specific ceRNA network contained 516 lncRNA nodes, 33 mRNA nodes, and 1050 edges (Figure 4B). Similar to the DLBCL-specific ceRNA network, one mRNA in the HL-specific ceRNA network may interact with two lncRNAs, and the average node degree of mRNAs was 1.97 (95% CI: 1.87–2.07).

True biological networks possess the important characteristic of being scale-free, which means that nodes with a higher degree are far fewer than those with a lower degree. We evaluated the node degree distributions of the two ceRNA networks. As shown in Figure 4C, the node degree distributions in both DLBCL- and HL-specific networks had long tails and fit the power law distribution, with *R*^2^ > 0.95, indicating that the two ceRNA networks were scale-free and similar to true biological networks.

### Functional Analysis of HL and DLBCL ceRNA Networks

In the DLBCL-specific ceRNA network, 18 out of 358 mRNAs were covered by the list of DLBCL-related genes, and 30 out of 533 mRNAs in the HL ceRNA network were reported to be associated with HL (Supplementary Table S2). In the DLBCL-specific ceRNA network, the mRNAs recorded in the DLBCL-related gene list had a higher degree, although a Mann–Whitney *U* test indicated that this tendency was not significant (*p* > 0.05). A similar tendency of miRNAs associated with HL was not found in HL-specific ceRNA network (Supplementary Figure S3).

Among the lncRNAs related to DLBCL and HL, only *MALAT1* and *GAS5* were involved in the DLBCL ceRNA network (*p* = 0.027), with a degree of 147 and 28, respectively. Interestingly, *HOTAIR*, which was associated with DLBCL, was involved in the HL ceRNA network, with a degree of 27. By contrast, *PVT1* was the only lncRNA recorded to be associated with HL, however, it was not included in the two ceRNA networks.

The pivot lncRNA, *MALAT1*, with the largest degree in both ceRNA networks, regulates the proliferation, invasion, and migration potential of metastatic tumor cells by sponging miR-195 in DLBCL (Isin et al., 2014; Wang et al., 2019). However, its regulatory function in HL has not yet been elucidated. LncRNAs associated with lymphoma, especially with HL, have been relatively less explored. The lncRNAs in the ceRNA networks may present promising candidate regulatory molecules.

### Network Analysis of ceRNA Subnetwork

**FIGURE 5:**
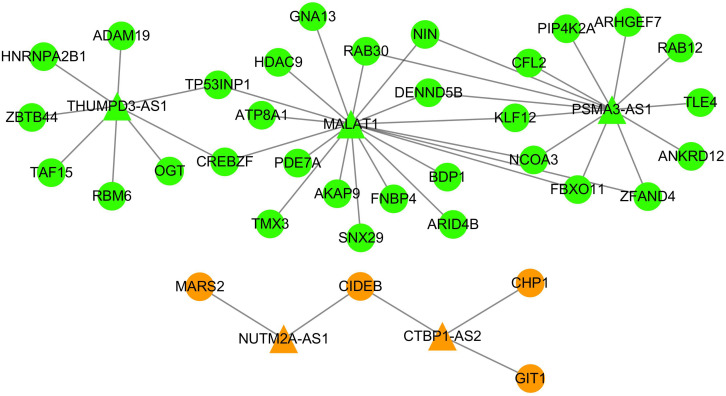
The ceRNA subnetwork between DLBCL and HL. The triangle nodes represent lncRNAs, and the circle nodes indicate mRNAs. The green nodes have the same direction of dysregulation in DLBCL and HL, while the orange nodes have the opposite regulation direction in these two types of lymphoma.

As shown in Figure 5, five lncRNAs (*MALAT1*, *CTBP1-AS*, *THUMPD3-AS*, *PSMA3-AS1*, and *NUTM2A-AS1*) were shared by DLBCL and HL ceRNA networks, which accounted for 20.83 and 31.25% of all lncRNAs corresponding to DLBCL and HL. By contrast, only 45 ceRNA pairs shared and occupied a minority of ceRNA networks (3.64% in DLBCL, 4.29% in HL). These results suggest that DLBCL and HL share a large proportion of lncRNAs involved in gene regulation, but that these lncRNAs have differing mRNA targets in their respective networks. The expression heatmaps of the shared ceRNAs are shown in Supplementary Figure S4.

Except for *MALAT1*, there was no evidence that the other four lncRNAs were associated with DLBCL or HL. However, it has been reported in previous studies that some of these lncRNAs are closely related to the development of other tumor types (Cui and Geng, 2019; Xu et al., 2019). For example, the overexpressed *CTBP1-AS* inhibits cell apoptosis and promotes tumorigenesis by sponging miRNA-940 in breast cancer (Cui and Geng, 2019).

Moreover, we searched the 35 mRNAs included in the ceRNA subnetwork in DisGeNET database and found that the majority of those mRNAs (31/35) were associated with the neoplastic process except for TMX3, SNX29, FNBP4, and RAB30. Among them, FBXO11, HDAC9, and GNA13 were biomarkers for DLBCL. Furthermore, HDAC9 was a biomarker for HL. These findings indicated that the lncRNAs and mRNAs in the ceRNA subnetwork represent common key molecules for the pathogenic processes involved in DLBCL and HL.

### Effect of mRNAs in the DLBCL-Specific ceRNA Network on Survival

**FIGURE 6:**
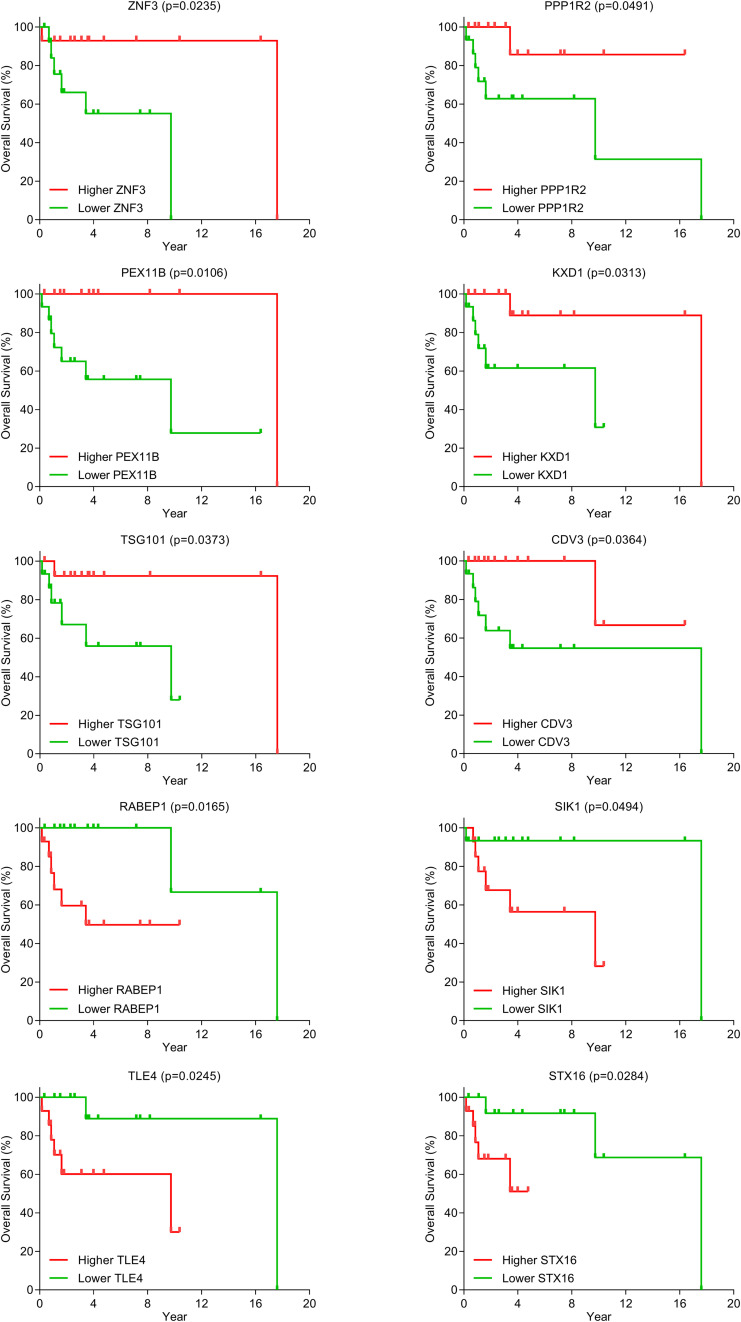
Kaplan–Meier curve analysis for mRNAs in the DLBCL ceRNA network.

Because only the clinical information and gene expression data of DLBCL patients are available from the TCGA database, we identified candidate biomarkers predictive of survival outcomes based on the DLBCL-specific ceRNA network. Kaplan–Meier curve analysis showed that 10 mRNAs (ZNF3, PPP1R2, RABEP1, PEX11B, SIK1, KXD1, TSG101, TLE4, CDV3, and STX16) were significantly differentially expressed (*p* < 0.05) (Figure 6). The groups with higher expression of ZNF3, PPP1R2, PEX11B, KXD1, TSG101, and CDV3 had better overall survival, while elevated expression of RABEP1, SIK1, TLE4, and STX16 was associated with poor prognosis.

**FIGURE 7:**
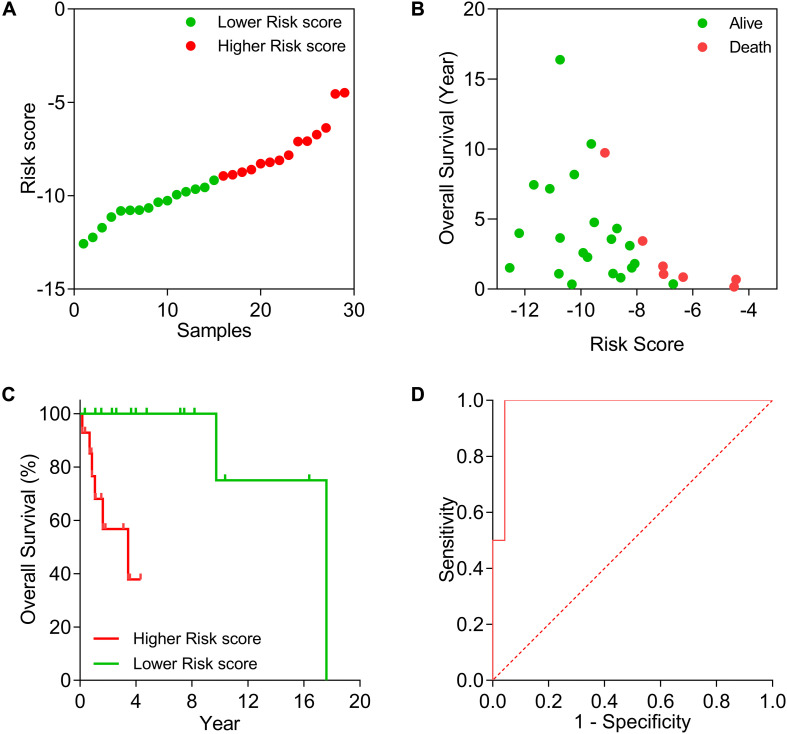
Evaluation of the prognostic risk model in DLBCL samples. **(A)** The distribution of survival risk. **(B)** The relationship between survival time and risk score. **(C)** Kaplan–Meier test of low-risk and high-risk groups. **(D)** ROC based on 5-year survival outcome.

We constructed a DLBCL survival risk model based on Lasso regression analysis. For each sample, a risk score analysis was conducted based on the model: risk score = −0.3905 × ZNF3 + 0.1006 × RABEP1 + 0.0961 × SIK1 − 0.259 ×KXD1 − 0.0835 × TSG101 + 0.2224 × TLE4 − 0.0524 × CDV3 + 0.0840 × STX16. The distribution of survival risk score and the relationship between survival time and risk score are shown in Figures 7A,B. The higher the risk score, the shorter the overall survival duration and the higher the death rate. Then, the DLBCL samples were divided into higher- and lower-risk score groups based on the median risk score (value = −8.9363). Results of Kaplan–Meier tests revealed that the low-risk group was associated with better overall survival (*p* = 0.0017) (Figure 7C). ROC analysis demonstrated that the AUC was 0.9783 (95% CI: 0.9300–1.0000) (Figure 7D). These results indicate that the prognostic model showed potentially good performance in terms of predicting the survival outcomes of DLBCL patients.

## Discussion

The ceRNA language is not dependent on a single protein-encoding mRNA, but instead focuses on the communication of mRNAs with other RNA categories, such as lncRNAs, in the transcriptome. Thus, the hypothesis offers a new layer of understanding into the complexity of tumorigenesis and progression. In this study, we systematically constructed DLBCL- and HL-specific ceRNA regulatory networks, which provide a theoretical basis for analyzing the regulatory functions of ceRNAs in DLBCL and HL.

We identified 10 mRNAs (ZNF3, PPP1R2, RABEP1, PEX11B, SIK1, KXD1, TSG101, TLE4, CDV3, and STX16) that were significantly correlated with overall survival in patients with DLBCL. Interestingly, although none of the 10 prognostic mRNAs in this work have been reported to be associated with lymphoma, some of them act as prognostic biomarkers in other malignant tumors (Yau et al., 2010; Andres et al., 2013; Liu et al., 2014; Wang et al., 2016; Xiao et al., 2018). For example, Yau et al. (Yau et al., 2010) have demonstrated that ZNF3 is a prognostic candidate gene in breast cancer. It has been reported that the overexpression of RABEP1, another biomarker of breast cancer, is predictive of reduced survival (Andres et al., 2013). Liu et al. (Liu et al., 2014) showed that TSG101 is an independent factor of poor prognosis in gallbladder cancer. Wang et al. (Wang et al., 2016) suggested that TLE4, which plays an important role in the development and progression of colorectal cancer, is a potential prognostic biomarker in this cancer. In addition, higher CDV3 expression is associated with poor survival rate in hepatocellular carcinoma (Xiao et al., 2018). Therefore, the 10 identified genes might represent promising prognostic biomarkers for DLBCL.

We were only able to identify a few DLBCL-related lncRNAs. The reasons for this may be as follows: (1) Some probes mapping to lncRNAs were filtered because they also mapped to other genes: for instance, the 225155_at probe mapped to SNHG5, SNORD50A, and SNORD50B; therefore, we removed this probe. (2) Because the array platform was designed to detect coding RNAs, some key lncRNAs, such as lincRNA-p21 and PANDAR, were not included. (3) Some lncRNAs were not significantly differentially expressed between lymphoma and control samples, such as TUG1 and PEG10. Despite these limitations, the study provided a general landscape of the ceRNA regulation models in DLBCL and HL.

In summary, in the present study, we constructed DLBCL- and HL-specific ceRNA networks and identified indirect interactions between lncRNAs and mRNAs. The results provide a method to identify lncRNA and mRNA biomarkers and further elucidate the mechanisms underlying the pathogenesis of DLBCL and HL.

## Data Availability Statement

All datasets presented in this study are included in the article/Supplementary Material.

## Author Contributions

JH conceived and designed this study. JK and QT wrote the manuscript. PY and YW analyzed the data. YZ drew the figures. All authors contributed to the article and approved the submitted version.

## Conflict of Interest

The authors declare that the research was conducted in the absence of any commercial or financial relationships that could be construed as a potential conflict of interest.

## Footnotes

1 http://metascape.org
