# Supplementary material for: Systematic Analysis of Competing Endogenous RNA Networks in Diffuse Large B-Cell Lymphoma and Hodgkin’s Lymphoma
Source: Front Genet. 2020 Sep 30;11:586688. doi: 10.3389/fgene.2020.586688 (PMC7554339; doi:10.3389/fgene.2020.586688)
Supplement: Supplementary file 1 [file Data_Sheet_1.docx]

Supplementary Material

# Supplementary Tables

Supplementary Table S1 The list of lncRNA associated with DLBCL and HL.

| lncRNA | Type | LncRNADisease | lnc2Cancer | MNDR |
| --- | --- | --- | --- | --- |
| *GAS5* | DLBCL | √ |  | √ |
| *PVT1* | DLBCL | √ | √ | √ |
| *SNHG5* | DLBCL | √ |  | √ |
| *HOTAIR* | DLBCL | √ | √ | √ |
| *HULC* | DLBCL | √ | √ | √ |
| *LUNAR1* | DLBCL | √ | √ | √ |
| *LOC283177* | DLBCL | √ |  | √ |
| *FAS-AS1* | DLBCL |  | √ |  |
| *lincRNA-p21* | DLBCL |  | √ | √ |
| *MALAT1* | DLBCL |  | √ | √ |
| *MINCR* | DLBCL |  | √ |  |
| *PANDAR* | DLBCL |  | √ |  |
| *PEG10* | DLBCL |  | √ | √ |
| *TUG1* | DLBCL |  |  | √ |
| *C13orf25* | DLBCL |  |  | √ |
| *PVT1* | HL | √ |  | √ |

Supplementary Table S2 DLBCL and HL-related genes contained in the DLBCL and HL specific ceRNA networks.

| CeRNA network | GeneSymbol | GeneID | Degree | GeneSymbol | GeneID | Degree |
| --- | --- | --- | --- | --- | --- | --- |
| DLBCL-specific ceRNA network | EIF4B | 1975 | 10 | HDAC9 | 9734 | 3 |
|  | KMT2D | 8085 | 9 | KMT2A | 4297 | 3 |
|  | FOXO1 | 2308 | 8 | MDM4 | 4194 | 2 |
|  | CALM1 | 801 | 8 | GNA13 | 10672 | 1 |
|  | SPEN | 23013 | 4 | TP53 | 7157 | 1 |
|  | MCL1 | 4170 | 4 | ARHGEF7 | 8874 | 1 |
|  | UHMK1 | 127933 | 4 | PER2 | 8864 | 1 |
|  | FBXO11 | 80204 | 3 | HIF1A | 3091 | 1 |
|  | ATM | 472 | 3 | MLLT10 | 8028 | 1 |
| HL-specific ceRNA network | RAPH1 | 65059 | 5 | IL15 | 3600 | 2 |
|  | LPP | 4026 | 4 | BCL2 | 596 | 1 |
|  | PTEN | 5728 | 4 | FAS | 355 | 1 |
|  | BCL6 | 604 | 3 | IL1A | 3552 | 1 |
|  | GCSAM | 257144 | 3 | IRF4 | 3662 | 1 |
|  | HDAC9 | 9734 | 3 | FGF2 | 2247 | 1 |
|  | CBFA2T3 | 863 | 3 | SNAP25 | 6616 | 1 |
|  | TSC1 | 7248 | 3 | CCR7 | 1236 | 1 |
|  | ZHX2 | 22882 | 2 | ETS1 | 2113 | 1 |
|  | TIMP2 | 7077 | 2 | HIF1A | 3091 | 1 |
|  | MAP3K8 | 1326 | 2 | BCL11A | 53335 | 1 |
|  | ELK3 | 2004 | 2 | CSF1 | 1435 | 1 |
|  | JAK1 | 3716 | 2 | SIX1 | 6495 | 1 |
|  | ARHGEF7 | 8874 | 2 | ARF1 | 375 | 1 |
|  | SLC40A1 | 30061 | 2 | IGF1R | 3480 | 1 |

# Supplementary Figures


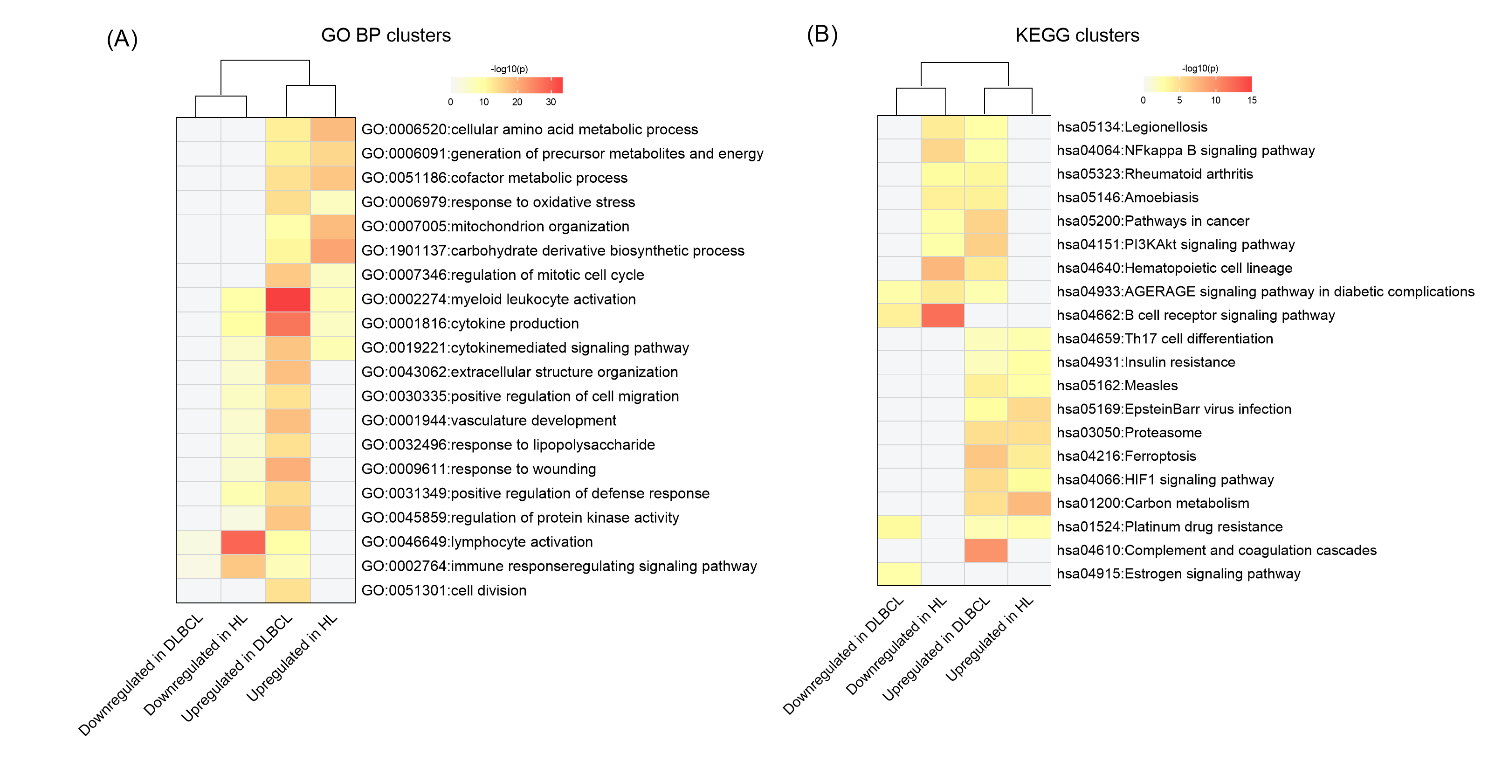


Supplementary Figure S1 The heatmaps of top 20 enriched GO and KEGG clusters by differentially expressed mRNAs.


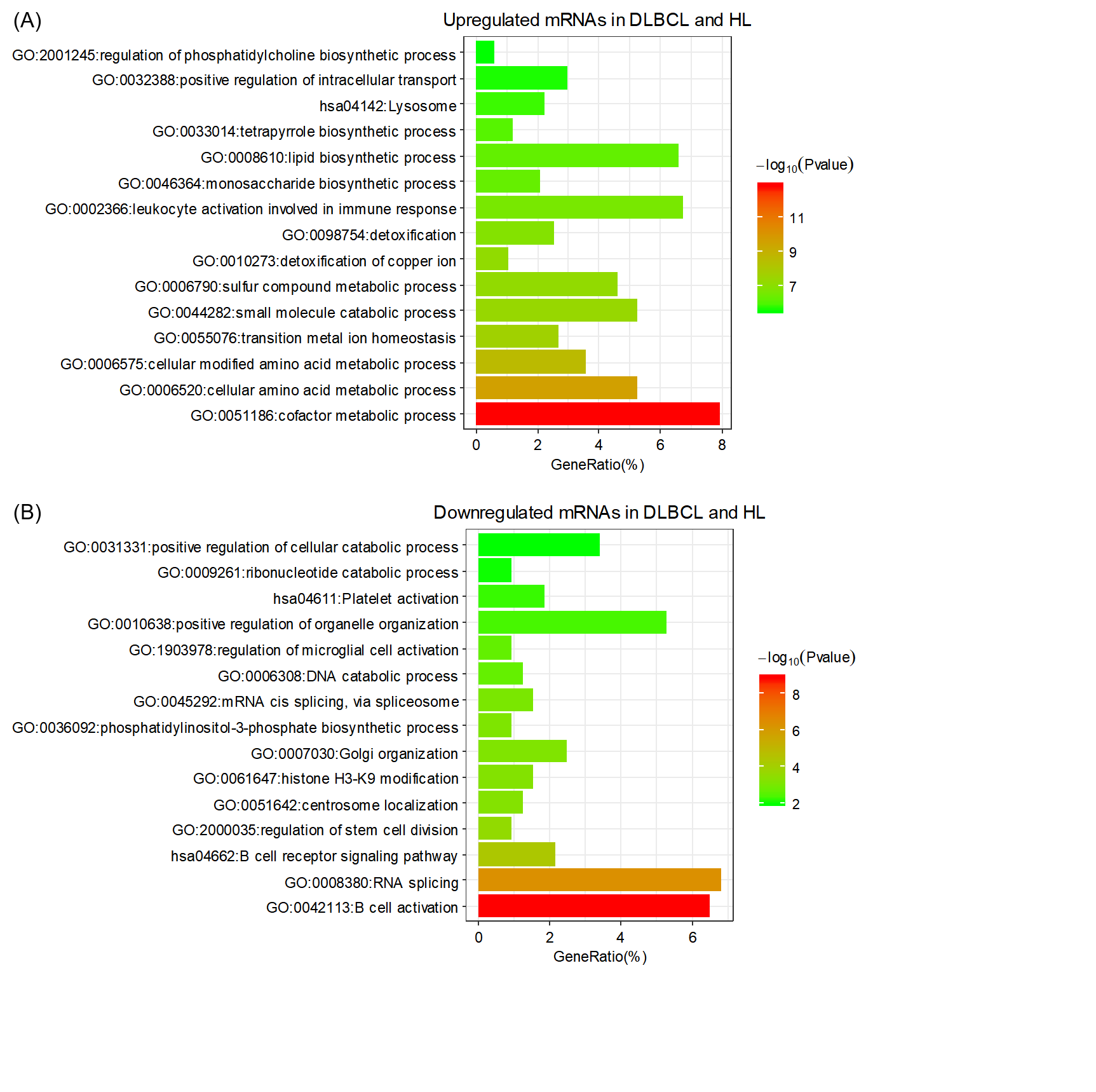


**Supplementary Figure S2** The top 15 clusters of enrichment pathways and biological processes of commonly dysregulated mRNAs in DLBCL and HL. (A) The top 15 clusters of enrichment pathways and biological processes of commonly upregulated mRNAs in DLBCL and HL. (B) The top 15 clusters of enrichment pathways and biological processes of commonly downregulated mRNAs in DLBCL and HL.


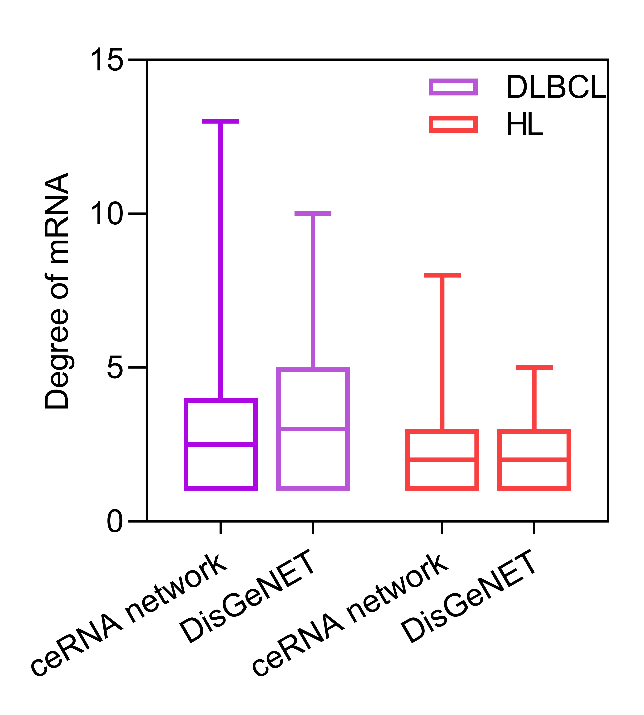


**Supplementary Figure 3** The degree comparisons of all mRNAs with lymphoma related mRNAs in ceRNA networks.


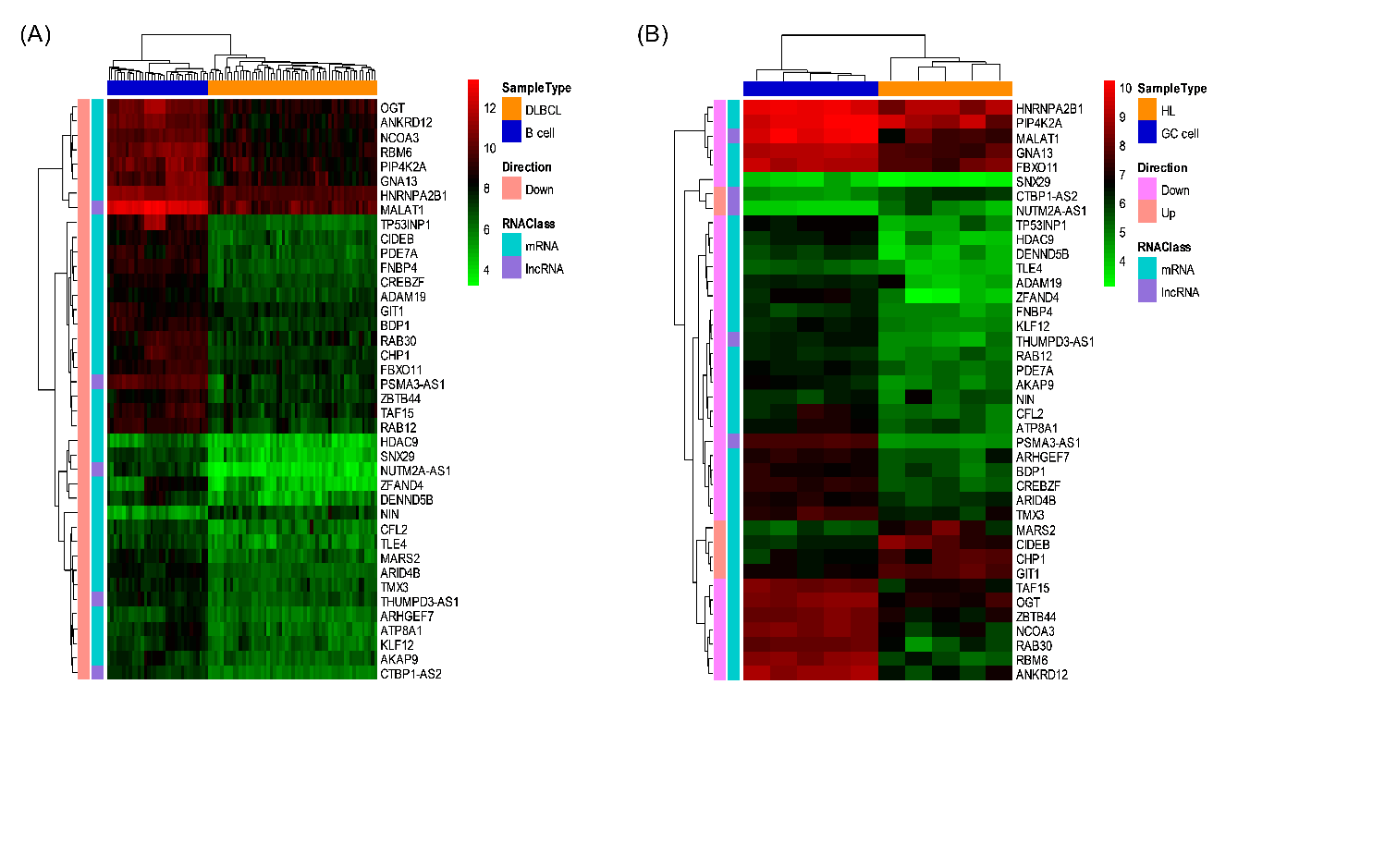


**Supplementary Figure 4** The expression heatmaps of ceRNAs in common ceRNA subnetwork. (A) The expression heatmap of common ceRNAs in DLBCL. (B) The expression heatmap of common ceRNAs in HL.
